# THEORIZING FORTITUDE: PERSPECTIVES FROM GSA’S ARTS, HUMANITIES, AND CULTURAL GERONTOLOGY ADVISORY PANEL

**DOI:** 10.1093/geroni/igae098.1979

**Published:** 2024-12-31

**Authors:** Sarah McKiddy, Katherine Ritchey, Ulla Kriebernegg

**Affiliations:** Chair; Co-Chair; Discussant

## Abstract

This symposium explores the impact of arts and humanities on cultural narratives of aging. Through the lens of theory-based examples, the authors argue for reaching beyond orthodox engines of gerontological exploration, such as medical research and the social sciences, to develop a more nuanced gerontology. However, moving beyond the arts-as-intervention model is not without pitfalls. Here, theoretical explorations challenge conventional ways of knowing by offering new insights into how cultural narratives of aging can be interpreted and shaped through the lens of the arts and humanities from a theoretical standpoint. Ultimately, the symposium suggests that fortitude, that is, courage in the face of adversity, resonates both as we travel through life facing our own challenges, and as the field of gerontology seeks to understand diverse human experiences of aging.

